# Fractures Associated with Metabolic Bone Disease in Extremely Preterm and Extremely Low Birth Weight Infants Before and After a Bone Health Program

**DOI:** 10.3390/children12111574

**Published:** 2025-11-19

**Authors:** Saif Alsaif, Lina Alsherbini, Talal Aljarbou, Manal Alshareef, Kamal Ali

**Affiliations:** 1 Neonatal Intensive Care Department, King Abdulaziz Medical City-Riyadh, Ministry of National Guard Health Affairs, Riyadh 11426, Saudi Arabia; saifs@mngha.med.sa (S.A.); alsherbinili@mngha.med.sa (L.A.); aljarbouta@mngha.med.sa (T.A.); alshareefma1@mngha.med.sa (M.A.); 2 King Abdullah International Medical Research Center, Riyadh 11481, Saudi Arabia; 3 College of Medicine, King Saud Bin Abdulaziz University for Health Sciences, Riyadh 11481, Saudi Arabia

**Keywords:** prematurity, metabolic bone disease, fractures, alkaline phosphatase, parathyroid hormone, length of stay, neonatal intensive care, quality improvement

## Abstract

**Highlights:**

**What are the main findings?**

- Fracture incidence decreased from 9.5% to 1.64% after program implementation (RR 0.17; NNT ≈ 13).
- Among infants who fractured, post-program cases had lower peak ALP/PTH, earlier detection, and shorter LOS.

**What is the implication of the main finding?**

- A protocolized bone health bundle can reduce fractures in extremely preterm/ELBW infants and is feasible for routine NICU practice.
- Results support multicenter evaluation to confirm generalizability, define core bundle components, and set quality benchmarks.

**Abstract:**

Background: Metabolic bone disease (MBD) of prematurity predisposes extremely preterm and extremely low birth weight (ELBW) infants to atraumatic fractures. Evidence on fracture reduction after structured Bone Health Programs (BHPs) remains limited. Methods: We conducted a single-center retrospective cohort of NICU admissions (2014–2024) with gestational age < 28 weeks and/or birth weight < 1000 g, comparing a pre-program era with a standardized BHP that incorporated protocolized biochemical surveillance, a week 4 screening radiograph, optimized mineral targets, pharmacist review of parenteral minerals, and “handle-with-care” practices. The study aimed to evaluate whether implementation of a structured BHP reduced fracture incidence and improved biochemical and clinical outcomes in extremely preterm and ELBW infants. Prespecified effect measures were risk ratio (RR), risk difference (RD) with 95% confidence intervals, Fisher’s exact *p* values, and number needed to treat (NNT). Among infants with fractures, we compared clinical course and biochemical context across eras. Results: Of 708 eligible infants, 221 were born pre-program and 487 post-program with similar baseline characteristics. Fracture incidence decreased from 9.5% (21/221) to 1.64% (8/487); RR 0.17 (95% CI 0.08–0.38); RD −7.86 percentage points; *p* < 0.001; NNT ≈ 13. Among infants who fractured, length of stay was lower post-program (104.1 ± 28.3 vs. 172.0 ± 91.5 days). Peak alkaline phosphatase and parathyroid hormone were also lower in the post-program era (ALP 501.3 ± 71.2 vs. 972.5 ± 93.5 IU/L, *p* = 0.032; PTH 23.1 ± 12.5 vs. 38.4 ± 21.7 pmol/L, *p* = 0.027), whereas serum phosphate and 25 OH vitamin D did not differ significantly. The fracture burden per infant decreased following the BHP (1.50 ± 0.53 vs. 3.19 ± 3.08, *p* = 0.024). Age at first fracture was earlier post-program, consistent with scheduled imaging (48.4 ± 34.9 vs. 83.9 ± 37.3 days, *p* = 0.031). Conclusions: A structured BHP was associated with a large reduction in fracture incidence and more favorable biochemical profiles, together with shorter hospitalization among fracture cases. Program elements that combine scheduled imaging, biochemical triggers, nutritional optimization, parenteral mineral stewardship, and standardized handling may improve skeletal outcomes. Multicenter prospective evaluations should confirm generalizability and define core components.

## 1. Introduction

Metabolic bone disease of prematurity arises from interrupted mineral accretion during the third trimester together with postnatal deficits in calcium and phosphate, leading to low bone mineral content and, in severe cases, fractures. Reported burden varies with definitions and the intensity of surveillance, but risk is highest in infants born before 28 weeks’ gestation or with birth weight under 1000 g [1]. In these infants, limited enteral tolerance, prolonged parenteral nutrition, suboptimal mineral delivery, and medications that alter mineral balance contribute to impaired bone mineralization and radiographic rickets [2,3,4,5,6].

Fractures in this population most often involve ribs and long bones and are linked to additional morbidity and longer hospitalization. Many are clinically silent and require radiographic confirmation, which makes structured surveillance important [2]. Risk is multifactorial, arising from an immature skeleton, suboptimal mineral intake, delayed fortification, medications that affect bone turnover, chronic inflammation, limited mobility, and overall illness severity; contemporary reviews and cohorts outline these contributors and their association with fracture outcomes in preterm infants [7]. As understanding of these mechanisms has grown, neonatal units have increasingly developed structured monitoring strategies to detect and prevent osteopenia-related fractures as part of comprehensive bone health initiatives.

Building on these prior reports, our study uniquely evaluates a structured Bone Health Program (BHP) implemented in a tertiary neonatal unit and quantifies its impact on fracture incidence in extremely preterm and extremely low birth weight infants. Several institutions have introduced structured pathways combining biochemical surveillance (for example, alkaline phosphatase and phosphate), nutrition protocols, imaging triggers, and “handle-with-care” bedside practices; early reports suggested reductions in osteopenia-related fractures with such quality initiatives [8]. However, most studies emphasized biochemical abnormalities rather than confirmed fractures, used small samples, or lacked a clear pre-post evaluation of a defined BHP. Definitions and surveillance schedules also vary, limiting comparability. Our study addresses these gaps by reporting real-world outcomes from a standardized BHP that incorporated biochemical, nutritional, and radiologic surveillance, comparing fracture incidence, biochemical trends, and clinical profiles before and after program implementation.

## 2. Methods

### 2.1. Study Design and Setting

We performed a retrospective single-center cohort study in a tertiary-level neonatal intensive care unit (NICU). Two nonoverlapping eras were compared: a pre-program period (2014–2016) and a post-program period (2017–2024). Our NICU provides tertiary care for extremely preterm and extremely low birth weight infants. King Abdullah International Medical Research Center (KAIMRC) approved the study and waived parental consent due to the retrospective nature of the study and the use of deidentified patient data.

### 2.2. Eligibility

In both eras we included live-born infants admitted to the NICU who met either of the following: gestational age (GA) < 28 weeks or birth weight (BW) < 1000 g. We excluded infants with a diagnosed congenital bone disorder at birth and those with insufficient documentation to ascertain fracture status.

### 2.3. Bone Health Program (Post-Era)

The unit’s Bone Health Program standardized prevention, surveillance, and management of MBD in a single protocol. Beginning at four weeks of postnatal age, infants underwent a scheduled bone profile drawn on the same day that included alkaline phosphatase, phosphate, calcium, parathyroid hormone, and 25-hydroxyvitamin D; results were interpreted against protocol thresholds, and repeat testing was scheduled if values rose or clinical concern persisted. MBD was defined biochemically by elevated alkaline phosphatase (>500 U/L) and/or elevated parathyroid hormone (>18 pmol/L), in conjunction with low serum phosphate or radiologic osteopenia when present. All radiographs were acquired on the NICU portable digital radiography system with tight collimation to the chest, abdomen, and proximal long bones, following ALARA principles (as low as reasonably achievable). Using equipment size-specific factors and published conversion coefficients for neonatal chest-abdomen radiography, the effective dose per week 4 babygram was approximately 0.02 to 0.06 mSv. The console displayed DAP (dose-area product) at acquisition; however, DAP values were not archived in the PACS (picture archiving and communication system) during the study period. Image quality control followed reject-repeat rules to minimize repeat exposures. Repeat radiographs were obtained if biochemical markers rose or if new signs suggested fracture, such as limb swelling or tenderness, crepitus, reduced movement or pseudo paralysis, deformity, or unexplained irritability on handling. Osteopenia was diagnosed with standardized AP babygrams using the following predefined criteria: diffuse reduction in bone mineralization with cortical thinning and loss of trabecular definition; supportive signs included metaphyseal lucencies or cupping and any rib or long-bone fractures. During the post-program era, nutrition practice followed the ESPGHAN recommendations available at the time and other contemporaneous neonatal nutrition guidance. The 2023 ESPGHAN position paper is cited to contextualize current standards and was not applied retrospectively [9,10]. Parenteral nutrition was started on day 1 and routinely included calcium and phosphate; solutions were compounded to maintain a molar calcium-to-phosphate ratio of approximately 1.3–1.7:1, with adjustments based on serum values and solubility limits, and prescriptions were pharmacist-reviewed for compatibility. Upon reaching full enteral feeds, infants were transitioned to oral calcium and phosphate supplementation with a molar ratio of 1.4–1.8:1, and vitamin D was provided routinely per unit protocol. At the bedside, a handle-with-care bundle minimized mechanical stress during positioning, procedures, and transfers, and staff education covered risk recognition, surveillance schedules, and clear escalation pathways.

### 2.4. Pre-Era Practice and Fracture Ascertainment

Before the BHP there was no standard biochemical or radiological screening for MBD. Laboratory tests (alkaline phosphatase, phosphate, calcium, parathyroid hormone, and 25-hydroxyvitamin D) were ordered at clinician discretion without preset thresholds or schedules. Routine screening babygrams were not performed. Radiographs were obtained for other clinical reasons (for example, line or tube checks, respiratory evaluation, or focal limb concerns), and fractures were identified either incidentally or after clinical triggers such as new limb swelling, focal tenderness, crepitus, reduced movement or pseudo paralysis, or visible or palpable deformity. All pre-era fractures were confirmed from contemporaneous official radiology reports archived in the institutional PACS and issued by consultant radiologists at the time of imaging. These reports served as the reference standard for fracture ascertainment in the pre-program era. A blinded re-review of pre-era images was not performed due to the retrospective design and time interval between study eras.

Parenteral nutrition was compounded according to contemporaneous unit practice; a standardized calcium-to-phosphate molar target was not in place and ratios were not routinely monitored, and enteral mineral supplementation followed clinician preference rather than protocol ranges. A structured “handle-with-care” bundle was not implemented and adherence to handling precautions was not monitored.

### 2.5. Data Sources and Variables

Eligible infants were identified from NICU admission logs and the electronic medical record. Fracture status during the index admission was ascertained from contemporaneous radiology reports and verified against the imaging archive. For the full cohort we abstracted gestational age at birth, birth weight, sex, small-for-gestational-age status, antenatal corticosteroid exposure, and mode of delivery; we also recorded the hospital length of stay.

For infants with one or more radiographically confirmed fractures, we captured the postnatal age at the first fracture, anatomical site, and the number of fractures documented during the admission. Biochemical context for fracture cases included alkaline phosphatase and parathyroid hormone values closest in time to the index fracture, together with the temporally nearest serum phosphate and 25-hydroxyvitamin D; when multiple results were available around the event, we used the value closest to the fracture time. These variables were used to describe clinical course and to compare fracture cases between eras.

### 2.6. Outcomes and Definitions

The primary outcome was the incidence proportion of infants with at least one radiographically confirmed fracture during the index NICU admission in each era. Secondary outcomes were the number of fractures per infant, the anatomical distribution of fractures, and the postnatal age at first fracture. For infants with fractures, we also summarized biochemical context using the values nearest in time to the index fracture, and compared length of stay across eras. A fracture was any break documented by the reporting radiologist on NICU radiographs.

### 2.7. Statistical Analysis

Analyses were performed in IBM SPSS Statistics v26 (IBM Corp., Armonk, NY, USA). Continuous variables are reported as mean ± SD or median (IQR), as appropriate, and categorical variables as *n* (%). The primary analysis compared the incidence proportion of ≥ 1 radiographically confirmed fracture during the index admission between eras; we estimated the risk ratio (RR) and risk difference (RD) with 95% confidence intervals (CI), tested significance with two-sided Fisher exact tests, and calculated the number needed to treat (NNT) as 1/RD.

Baseline comparability for gestational age, birth weight, sex, small-for-gestational-age status, antenatal corticosteroids, and mode of delivery was assessed using Welch t tests or Mann–Whitney U tests for continuous variables and chi-square or Fisher exact tests for categorical variables. Among infants with fractures, we compared postnatal age at first fracture, number of fractures per infant, anatomical distribution, biochemical values closest to the index fracture (alkaline phosphatase, parathyroid hormone, phosphate, 25-hydroxyvitamin D), and length of stay using Welch *t* tests, Mann–Whitney U tests, chi-square tests, or Fisher exact tests as appropriate.

All tests were two-sided with α = 0.05. Analyses used complete-case data without imputation.

## 3. Results

**Figure 1:**
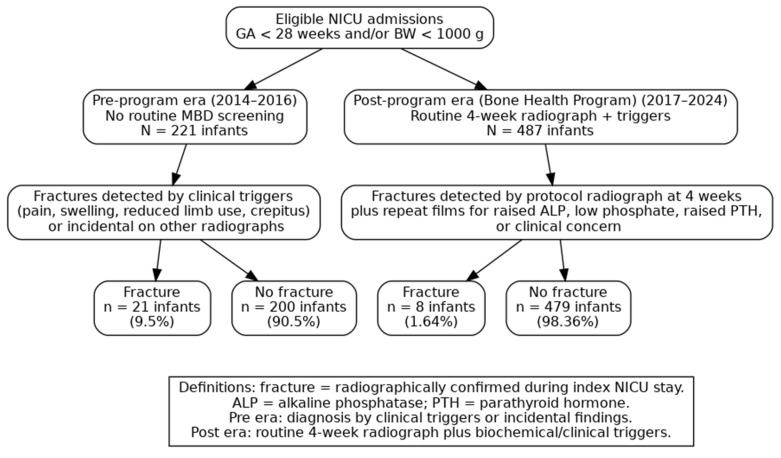
Study flow for fracture surveillance across eras.

Figure 1 presents the study flow and fracture outcomes across the two surveillance eras. In the pre-program era (2014–2016; *n* = 221 infants), 21 infants (9.5%) had at least one radiographically confirmed fracture and 200 (90.5%) had no fracture. In the post-program era (2017–2024; *n* = 487 infants) under the BHP, 8 infants (1.64%) had a fracture and 479 (98.36%) had no fracture.

Table 1 (baseline characteristics). The pre-program (*n* = 221) and post-program (*n* = 487) cohorts were closely comparable. Mean gestational age was 26.8 ± 2.3 vs. 26.9 ± 2.2 weeks (*p* = 0.64), and mean birth weight was 870 ± 210 vs. 878 ± 205 g (*p* = 0.58). Antenatal corticosteroid exposure (76.0% vs. 78.0%), male sex (53.8% vs. 55.0%), small-for-gestational-age status (26.2% vs. 24.0%), and cesarean delivery (63.3% vs. 65.1%) did not differ (all *p* > 0.05). These similarities indicate that differences in fracture outcomes are unlikely to be explained by baseline case mix.

Table 2 shows a marked reduction in fracture risk after the BHP. In the pre-program era, 21 of 221 infants had at least one radiographically confirmed fracture (9.5 percent, 95% CI 5.97 to 14.11). In the post-program era, 8 of 487 infants fractured (1.64 percent, 95% CI 0.71 to 3.20). This corresponds to a risk ratio of 0.17 (95% CI 0.08 to 0.38, *p* < 0.001), indicating an 83 percent relative reduction, and an absolute risk difference of −7.86 percentage points (95% CI −13.25 to −3.09, *p* < 0.001). The number needed to treat is approximately 13, meaning that managing 13 infants under the program would prevent 1 infant from developing a fracture compared with historical care. If the pre-program risk had persisted in the post-era, about 46 infants with fractures would have been expected among 487 infants, instead of the 8 infants that were observed.

Table 3 shows that infants who fractured in the pre- and post-eras had similar baseline gestational age and birth weight (both *p* > 0.50). After program implementation, several outcomes improved among fracture cases. The mean number of fractures per infant was lower (1.50 vs. 3.19, Δ −1.69, 95% CI −3.13 to −0.25; *p* = 0.024), and length of stay was shorter (104.1 vs. 172.0 days, Δ −67.9, 95% CI −113.7 to −22.0; *p* = 0.005). Peak ALP and PTH measured nearest to the index fracture were reduced in the post era (ALP 501 vs. 972 U/L, Δ −471, 95% CI −899 to −43; *p* = 0.032; PTH 23.1 vs. 38.4 pmol/L, Δ −15.3, 95% CI −28.7 to −1.9; *p* = 0.027), whereas serum phosphate and 25-OH vitamin D did not differ (both *p* > 0.30). The age at first fracture occurred earlier post-program (48.4 vs. 83.9 days, Δ −35.5, 95% CI −67.4 to −3.7; *p* = 0.031), consistent with routine week 4 screening and earlier recognition.

**Figure 2:**
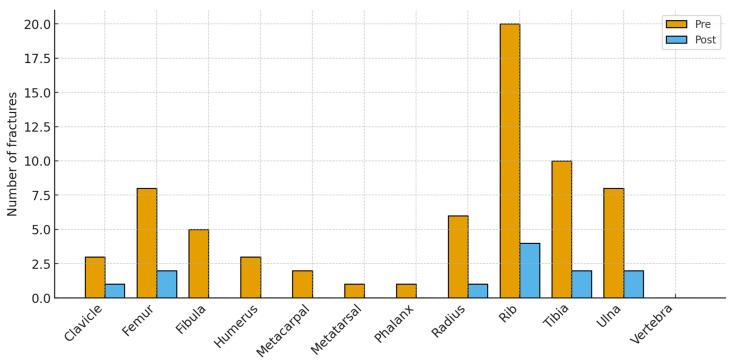
Fracture sites before and after the Bone Health Program.

Figure 2 shows the anatomical distribution of fractures before and after the BHP. Bars display the number of fractured bones at each site; totals can exceed the number of infants because some sustained multiple fractures. Pre-program, fractures clustered in the ribs (20 of 67 total fractures, ~30%) and in long bones (tibia 10, ulna 8, femur 8, radius 6). Smaller numbers occurred at the fibula 5, humerus 3, clavicle 3, metacarpals 2, metatarsals 1, and phalanges 1; no vertebral fractures were recorded. Post-program, totals fell across nearly all sites (12 fractures in total): ribs 4, tibia 2, ulna 2, femur 2, radius 1, clavicle 1, with no other sites affected. The largest absolute reductions were at the ribs (−16), tibia (−8), ulna (−6), and femur (−6), indicating a broad reduction in fracture burden while preserving the typical neonatal pattern of rib and long-bone involvement at much lower frequency.

## 4. Discussion

This single-center retrospective cohort compared two eras in a tertiary-level NICU to evaluate whether a structured BHP was associated with fewer radiographically confirmed fractures in extremely preterm and/or extremely low birth weight infants. The program incorporated protocolized biochemical surveillance, a screening radiograph at four weeks, nutrition targets, and unit-wide safe handling practices. Fracture incidence decreased from 9.5% (21/221) before the program to 1.64% (8/487) after the program, with a risk ratio of 0.17 and an absolute risk reduction of 7.86 percentage points. Among infants who fractured, length of stay fell by about 68 days on average, peak alkaline phosphatase and parathyroid hormone were lower, fracture burden per infant was smaller, and fractures were detected earlier in postnatal life, consistent with scheduled screening. Baseline characteristics were similar across eras.

The pattern of results suggests that the BHP influenced both detection and disease severity. Earlier identification after implementation is expected given the week 4 radiograph and laboratory triggers; the smaller number of fractures per infant and the lower length of stay among fracture cases indicate a clinically meaningful reduction in skeletal morbidity. The decrease in peak alkaline phosphatase and parathyroid hormone is biologically coherent with improved mineral delivery and better bone turnover control in the post-program era. Reports in the literature support the use of serial alkaline phosphatase as a practical surveillance tool and note threshold ranges that prompt imaging, typically around 700–800 IU/L, with some studies showing risk signals above 500 IU/L in very immature infants [11]. This consistency supports our interpretation that the observed biochemical improvements reflect more effective mineral supplementation and earlier detection of evolving osteopenia, aligning with prior evidence for threshold-guided monitoring strategies.

Our effect size is clinically large and actionable. The fracture risk fell from 9.5% to 1.64% (risk ratio 0.17), which is an 83 percent relative reduction and an absolute risk difference of 7.86 percentage points. The number needed to treat is about 13, so for every 13 extremely preterm or extremely low birth weight infants managed under the program, 1 infant avoids a fracture. This NNT is favorable for a neonatal quality bundle that is based on standardized workflow, surveillance, and nutrition rather than new equipment. The direction of potential detection bias argues against an artifact: adding a week 4 radiograph and biochemical triggers would be expected to identify more early fractures, not fewer, yet the risk decreased and fractures were recognized earlier. The concurrent reductions in peak alkaline phosphatase and parathyroid hormone among fracture cases support a biological mechanism of improved mineral delivery and better control of bone turnover. The largest absolute reductions at the ribs and long bones also fit a prevention effect because these sites are most sensitive to mineral deficits and handling forces. Applied at service level, the observed absolute risk reduction corresponds to preventing an estimated 12 infants with fractures per 150 eligible infants per year, assuming case mix and baseline risk resemble our pre-program era, and the protocol is implemented consistently. Although residual confounding and era effects cannot be excluded, the consistency across metrics, including lower risk, fewer fractures per infant, earlier recognition, and improved biochemistry, makes a causal contribution from the program likely. Multicenter implementation studies could refine which components drive the effect and whether similar NNT values are achievable in other NICU settings.

A review of prior work shows wide variation in fracture rates associated with MBD in preterm infants, reflecting differences in case definitions, surveillance intensity, and baseline risk. In a prospective VLBW cohort, Koo and colleagues reported radiographic rickets and/or fractures in about one quarter of infants on follow-up, indicating substantial skeletal morbidity among higher-risk infants [12]. Backström’s narrative review described radiographic rickets in 55% of infants <1000 g and 23% of those <1500 g, with fractures in about 24% of <1500 g infants, consistent with high prevalence when mineral deficits are pronounced [13]. In an ELBW cohort, Viswanathan et al. found radiographic MBD in 31% and fractures in 24 of 230 infants (≈10.4% overall; 33.8% among those with MBD) [4]. Other population-based or unit-level series have reported lower rates in broader preterm populations: Dabezies and Warren observed 26 fractures among 247 VLBW infants (10.5%) with nutritional rickets identified during NICU care [5]; Amir et al. reported non-traumatic fractures in about 1.2% of premature infants [14]; Lucas-Herald et al. documented center-specific rib fracture prevalences of 2.9%, 0.5%, and 0.4% in ex-preterm cohorts [15]; and Wei et al. estimated an in-NICU fracture incidence of about 1.6% in a single-center series, noting that not all cases were spontaneous or attributable to MBD [16]. In this context, our pre-program fracture risk of 9.5% (21/221) lies within the range reported for very preterm and ELBW cohorts and is close to the 10–11% observed in unit-based series with targeted radiography, whereas our post-program risk of 1.64% (8/487) is toward the lower end of published estimates and approaches rates from broader preterm series that used routine screening and standardized handling. The Bone Health Program likely reduced missed subclinical disease and enabled earlier nutritional and caregiving interventions that limited subsequent fragility. Differences in inclusion criteria, exclusion of traumatic fractures, and the larger denominator in the post-program era may also contribute to the lower observed incidence, but the magnitude and internal consistency of reduction support a true improvement in bone health.

Beyond our program’s biomarker improvements, external evidence clarifies which tests and thresholds should trigger imaging or nutritional escalation. Comparative data indicate that PTH complements ALP for detecting clinically important MBD in very preterm infants; in one cohort, elevated PTH identified infants at risk of severe disease when ALP alone was insufficient, supporting the combined-marker approach used in our post-program era [17]. Studies of serial ALP show its value as an early indicator of radiographic osteopenia, consistent with our finding that scheduled testing shifted detection earlier without evidence of overtreatment [18]. Screening reviews summarize these findings and recommend structured pathways that pair biochemical triggers with targeted radiography, which aligns with our BHP [19]. Nutritional work also informs implementation: a randomized trial shows that 800 IU/day versus 400 IU/day vitamin D reduces biochemical deficiency without toxicity, yet effects on bone mineral content are modest, supporting the view that adequate calcium and phosphate delivery remains the main driver of skeletal outcomes [20]. Because parenteral solutions need to meet calcium–phosphate compatibility limits, laboratory and clinical studies describe precipitation risk and the influence of ratios, amino acid concentration, and temperature, which justifies the pharmacist review embedded in our protocol [21]. Finally, dual-energy X-ray absorptiometry (DXA) and quantitative ultrasound (QUS) are not practical for routine bedside screening, but validation studies show they can track mineralization trajectories for research or targeted follow-up, complementing rather than replacing a biochemistry-plus-radiography pathway in the NICU [22].

The study has several strengths and some limitations. Strengths include a large, contemporary cohort focused on extremely preterm and/or extremely low birth weight infants; a clear, radiology-based fracture outcome applied across eras; prespecified risk comparison with effect estimates (risk ratio, risk difference, number needed to treat); and consistent secondary signals among fracture cases (fewer fractures per infant, shorter length of stay, lower alkaline phosphatase and parathyroid hormone). Baseline characteristics were well matched between eras, reducing confounding by case mix. Although a routine week 4 radiograph was implemented as part of the BHP, subsequent imaging was performed only when clinically or biochemically indicated. This approach standardized rather than intensified radiographic surveillance. The consistent timing of this screening film, combined with selective follow-up, minimizes the likelihood of under-ascertainment. The reduction in fracture incidence thus reflects a true improvement in bone health outcomes rather than differences in imaging practices.

Limitations relate mainly to the retrospective, era-based design. Ascertainment differed between eras: the pre-era relied on symptom-driven or incidental imaging, whereas the post-era included scheduled week 4 radiographs and repeat films for biochemical or clinical triggers. This could shift detection toward earlier and milder disease after implementation; however, such a shift would be expected to increase, not decrease, the number of identified cases. The small number of post-era fracture events limits multivariable modeling and subgroup analyses. Secular changes in neonatal care that were not captured (for example, ventilation strategies or nutrition practices outside the program) may have contributed to the effect. Some exposures were not available with precision, such as cumulative diuretic or steroid dosing and exact mineral delivery in the pre-era. Quantitative ultrasound provides bedside, radiation-free assessment and shows moderate correlation with DXA, but limited reference ranges and motion or positioning issues in extremely preterm infants currently limit routine use [23]. We view QUS as a promising adjunct for future program updates when robust neonatal reference data becomes available. Finally, we did not evaluate pain, functional outcomes, or post-discharge fractures, and single-center practice may limit generalizability. Overall, the direction and magnitude of the findings across multiple measures support a real reduction in fracture risk, but confirmation in prospective, multicenter studies is warranted.

## 5. Conclusions

Implementation of a structured Bone Health Program was associated with a marked fall in fracture risk among extremely preterm and/or extremely low birth weight infants, from 9.5% pre-program to 1.64% post-program (risk ratio 0.17; number needed to treat ≈13). Among infants who fractured, the post-era also showed earlier detection, fewer fractures per infant, lower alkaline phosphatase and parathyroid hormone, and shorter hospitalization. These findings support a pragmatic bundle comprising a week 4 screening radiograph, routine biochemical surveillance with repeat imaging when indicated, optimized calcium and phosphate intake, and standardized safe handling practices. Confirmation in prospective multicenter studies is warranted.

## Figures and Tables

**Table 1:** Baseline characteristics before and after the Bone Health Program.

Characteristic	Pre-Program (N = 221)	Post-Program (N = 487)	*p* Value
Gestational age, weeks (mean ± SD)	26.8 ± 2.3	26.9 ± 2.2	0.64
Birth weight, g (mean ± SD)	870 ± 210	878 ± 205	0.58
Antenatal steroids, n/N (%)	168/221 (76.0)	380/487 (78.0)	0.49
Male sex, n/N (%)	119/221 (53.8)	268/487 (55.0)	0.77
Small-for-gestational-age, n/N (%)	58/221 (26.2)	117/487 (24.0)	0.49
Cesarean delivery, n/N (%)	140/221 (63.3)	317/487 (65.1)	0.66

Values are mean ± SD or n/N (%). *p* values from Welch’s *t* test (continuous variables) and χ^2^ (or Fisher’s exact test when expected counts were small) for categorical variables.

**Table 2:** Fracture risk before and after implementation of the Bone Health Program.

Group/Metric	Infants/N	Risk (%)	95% CI (%)	Definition	Estimate	95% CI	*p* Value ^†^
Pre-program	21/221	9.50	5.97–14.11	—	—	—	—
Post-program	8/487	1.64	0.71–3.20	—	—	—	—
Risk ratio	—	—	—	Post ÷ Pre	0.17	0.08–0.38	<0.001
Risk difference	—	—	—	Post − Pre (percentage points)	−7.86	−13.25 to −3.09	<0.001
Number needed to treat (NNT)	—	—	—	1 ÷ absolute RD	≈13	—	—

Risks are shown with 95% confidence intervals. Risk ratio and risk difference are reported with 95% confidence intervals. ^†^ two-sided Fisher’s exact test.

**Table 3:** Characteristics of infants with fractures before and after the Bone Health Program.

Variable	Pre (*n* = 21)	Post (*n* = 8)	Δ (Post–Pre)	95% CI for Δ	*p* Value
Gestational age (weeks)	227. 00 ± 3.13.	26.50 ± 1.51	0.50	−1.29 to 2.29	0.569
Birth weight (g)	873.10 ± 290.57	870.00 ± 131.91	3.10	−158.74 to 164.93	0.969
Length of stay (days)	172.00 ± 91.46	104.13 ± 28.29	−67.88	−113.72 to −22.03	0.005
Alkaline phosphatase (U/L)	972.48 ± 93.51	501.25 ± 71.18	−471.23	−899.04 to −43.41	0.032
Parathyroid hormone (pmol/L)	38.39 ± 21.74	23.11 ± 12.47	−15.28	−28.71 to −1.86	0.027
25-OH vitamin D (nmol/L)	55.24 ± 20.22	53.86 ± 5.17	−1.38	−11.21 to 8.46	0.776
Serum phosphate (mmol/L)	1.04 ± 0.24	0.95 ± 0.23	−0.09	−0.30 to 0.11	0.350
Number of fractures (per infant)	3.19 ± 3.08	1.50 ± 0.53	−1.69	−3.13 to −0.25	0.024
Age at first fracture (days)	83.90 ± 37.28	48.38 ± 34.93	−35.53	−67.36 to −3.70	0.031
Antenatal steroids, n/N (%)	14/21 (66.7)	6/8 (75.0)	+8.3 pp	—	1.000
Male sex, n/N (%)	13/21 (61.9)	4/8 (50.0)	−11.9 pp	—	0.683

Continuous variables are mean ± SD and compared with Welch’s *t* test; Δ denotes post minus pre with 95% confidence intervals. Categorical variables are n/N (%) and compared with Fisher’s exact test; Δ is the difference in percentage points (pp). Biochemical values are those closest in time to the index fracture for each infant.

## Data Availability

The data presented in this study are available on request from the corresponding author. Restrictions apply because the dataset contains patient-level clinical information and cannot be publicly shared to protect confidentiality.

## Abbreviations

The following abbreviations are used in this manuscript:

ALP	alkaline phosphatase
AAP	American Academy of Pediatrics
BHP	Bone health program
BW	birth weight
CI	confidence interval
DXA	dual-energy X-ray absorptiometry
ELBW	extremely low birth weight
ESPGHAN	European Society for Pediatric Gastroenterology, Hepatology, and Nutrition
GA	gestational age
IQR	interquartile range
LOS	length of stay
MBD	metabolic bone disease
NICU	neonatal intensive care unit
NNT	number needed to treat
PTH	parathyroid hormone
RD	risk difference
RR	risk ratio
SD	standard deviation
SGA	small-for-gestational-age
25-OH vitamin D	25-hydroxyvitamin D
